# Role of TRAP1 and estrogen receptor alpha in patients with ovarian cancer -A study of the OVCAD consortium

**DOI:** 10.1186/1476-4598-11-69

**Published:** 2012-09-14

**Authors:** Stefanie Aust, Anna Bachmayr-Heyda, Petra Pateisky, Dan Tong, Silvia Darb-Esfahani, Carsten Denkert, Radoslav Chekerov, Jalid Sehouli, Sven Mahner, Toon Van Gorp, Ignace Vergote, Paul Speiser, Reinhard Horvat, Robert Zeillinger, Dietmar Pils

**Affiliations:** 1 Department of Obstetrics and Gynecology Molecular Oncology Group, Medical University of Vienna, Vienna, Austria; 2 Department of Gynecology Campus Virchow Klinikum, Charite Medical University, Berlin, Germany; 3 Department of Gynecology and Gynecologic Oncology, University Medical Center Hamburg-Eppendorf, Hamburg, Germany; 4 Department of Obstetrics and Gynecology, University Hospitals Leuven, Katholieke Universiteit Leuven, Leuven, Belgium; 5 Division of Gynaecological Oncology, Department of Obstetrics and Gynaecology, MUMC+ GROW School for Oncology and Developmental Biology, PO Box 5800, Maastricht, 6202AZ, The Netherlands; 6 Clinical Institute of Pathology, Medical University of Vienna, Vienna, Austria

**Keywords:** TRAP1, Estrogen receptor, Immunohistochemistry, Prognosis, Ovarian cancer

## Abstract

**Background:**

The role of the tumor necrosis factor receptor associated protein 1 (TRAP1) – supposed to be involved in protection of cells from apoptosis and oxidative stress – has just started to be investigated in ovarian cancer. TRAP1 has been shown to be estrogen up-regulated in estrogen receptor α (ERα) positive ovarian cancer cells. The clinical impact of TRAP1 is not clear so far and the significance of ERα expression as therapeutic and prognostic marker is still controversial. Therefore, we investigated the importance of TRAP1 together with ERα in regard to clinicopathological parameters, chemotherapy response, and survival.

**Methods and results:**

Expressions of TRAP1 and ERα were evaluated by immunohistochemical staining of tissue microarrays comprised of 208 ovarian cancer samples. TRAP1 was highly expressed in 55% and ERα was expressed in 52% of all cases. High TRAP1 expression correlated significantly with ERα (p < 0.001) but high TRAP1 expression was also found in 42% of ERα negative cases. High TRAP1 expression correlated significantly with favorable chemotherapy-response (HR = 0.48; 95%CI 0.24-0.96, p=0.037) and showed a significant impact on overall survival (OS) (HR = 0.65; 95%CI 0.43-0.99, p = 0.044). ERα expression was a favorable prognostic factor for OS in univariate and multivariate analyses. Interestingly, the combined pattern (ERα positive and/or TRAP1-high) revealed the strongest independent and significant positive influence on OS (HR = 0.41; 95%CI 0.27-0.64).

**Conclusion:**

Immunohistochemical evaluation of TRAP1 together with ERα provides significant prognostic information. TRAP1 alone is significantly associated with chemotherapy response and overall survival, rendering TRAP1 as interesting scientific and therapeutic target.

## Introduction

Molecular chaperones of the Hsp90 (90-kDa heat shock protein) family are involved in cancer development and malignant progression. TRAP1/Hsp75 (tumor necrosis factor receptor associated protein 1), a paralogue of the Hsp90 family, has been recently described as a molecular marker and novel therapeutic target in local and metastatic prostate cancer [1]. Increased expression of TRAP1 in multidrug resistant colorectal cancer was suggested to favor chemotherapy resistance [2]. In breast cancer cells, HSPs influence tumorigenesis [3] and in ovarian cancer, TRAP1 has recently been questioned as a new potential molecular target [4].

Ovarian cancer is the leading cause of death from gynecologic malignancies in western countries, whereby peritoneal metastasis and chemotherapy resistance as well as relapse after chemotherapy remain scientific and clinical challenges. Trying to understand the mechanisms involved in cancer progression and chemotherapy resistance in epithelial ovarian carcinoma (EOC), the role of heat shock proteins, including TRAP1, has just started to be investigated [4-6].

Chemotherapy effectiveness depends to a high amount on the ability of ovarian cancer cells to undergo drug-induced apoptosis [7]. TRAP1, described to be involved in apoptosis evasion, was observed to be significantly up-regulated in Cisplatin resistant ovarian tumor cell lines [8]. Microarray analysis of expression changes in human ERα-positive ovarian cancer cell lines upon 17ß-estradiol stimulation, identified TRAP1 to be estrogen up-regulated and to be involved in growth regulation of EOC [9]. In certain EOCs, high levels of ERα are frequently expressed at time of diagnosis and expression levels of ERα dependent up-regulated proteins such as TRAP1 were discussed to predict endocrine responsiveness [10].

However, the importance of ERα expression as therapeutic and prognostic marker in EOC is still controversial [11]. Prognostic significance of ERα expression was recently described as favorable, revealing a significantly longer overall survival [12-14]. However, in other studies this was not observed [15,16]. In young women, the expression of ER alone was not associated with favorable survival but the combination of ER+/PR + revealed a superior overall survival [17]. Additionally, superior prognosis was described in ER-/PR + invasive ovarian cancer [18] when compared to all other combinations. Thus, TRAP1 might become of relevance when predictors of endocrine therapy response in EOC will be further elucidated.

Up to now, only a limited number of studies have focused on this Hsp90 related molecule and most of the results were obtained from cell line models or mouse models [19,20]. TRAP1’s levels are consistently elevated in several human malignancies (i.e. colon, prostate, and nasopharyngeal carcinomas), while present at very low levels, and sometimes undetectable in the corresponding normal tissues. In EOC, a number of studies have investigated the association of differences in gene expression at time of primary surgery and therapy-response [21,22]. It has been demonstrated that estrogen-regulated gene expression can predict response to endocrine therapy with the aromatase inhibitor Letrozole in patients with ovarian cancer. The expression of estrogen-regulated genes such as TRAP1, TFF1, TFF3, TOP2A and UBE2C was significantly different between CA125 progressors and non-progressors in a sample of 42 patients after treatment with Letrozole, whereby TRAP1 was significantly increased in Letrozole-responsive patients [10].

The aim of our study was to further clarify the role of TRAP1 in a large and homogenous sample of human EOC in regard to clinicopathological parameters, therapy response, and patient outcome. We examined the pattern of TRAP1 expression in prospectively collected ovarian tumor tissue from 208 non-FIGO I patients collected in the course of the EU-project OVCAD (Ovarian Cancer: Diagnosis of a silent killer, no. 018698). To better understand the function of TRAP1 and to provide further insight into the relationship between ERα expression and tumor behavior as well as patients’ outcome, expression levels of ERα were analyzed as well and correlated with clinicopathological parameters.

## Materials and methods

### Patients cohort

Within the context of the FP6 EU-project OVCAD (http://www.ovcad.org), samples from primary EOC were prospectively collected at the University clinics of Berlin, Hamburg, Innsbruck, Leuven, and Vienna (OVCAD-consortium). Samples were collected according to standard operation procedures established in OVCAD. Clinical and histopathological data as well as follow-up data were collected by experienced clinicians. Patients presenting with benign ovarian diseases, FIGO I stage EOC or secondary malignant diseases were excluded. 85% of Patients were treated according to standards of the institutions involved with upfront surgery and adjuvant platinum–based chemotherapy. A total of 31 patients (14.9%) were treated with neoadjuvant chemotherapy (platinum–based) followed by an intervention-debulking and further adjuvant chemotherapy. Only patients with debulking surgery and platinum–based chemotherapy were included to the OVCAD patient cohort. The study protocol was approved by the Ethics Committees of the participating OVCAD partners. All patients gave pre-operative written informed consent before enrollment in the study. Overall survival (OS) was defined as the time interval between diagnosis and cancer correlated death and progression free survival (PFS) as the time between diagnosis and disease progression. Overall observation time was the time interval between diagnosis and last contact, defined as death or last follow-up. Therapy response to chemotherapy was defined according to the WHO criteria; i.e. progression of disease after first-line chemotherapy was defined by an increase in the nadir serum CA-125 level of at least two according to the GCIG criteria or by radiological confirmation. Patients were classified as non-responder if progression was diagnosed during treatment or recurrence within six months after end of first-line chemotherapy. Patients without recurrence, cancer progression or death were censored at the time of last follow-up. Experienced gynecological oncologists and pathologists performed the clinical and histopathological evaluation and the evaluation of response to first-line treatment.

### Immunostaining

Five tissue microarrays (TMAs) comprised of two tissue sections of 208 patients per patient were analyzed. Samples were collected between 2006 and 2008. Mitochondrial staining of monoclonal rabbit anti-TRAP1 antibody (1:1,000; Cat-Id/Clone-ID 3609-1/EPR5381, Epitomics, USA) was verified by a colocalization analysis employing double-immunofluorescence staining with mouse anti-COX5 antibody as mitochondrial marker (1:250; Invitrogen). The fluorescence labeled secondary antibodies, goat anti-rabbit (1:5,000; Invitrogen, AlexaFluor® 488 fragment of goat anti-rabbit IgG (H + L)) and goat anti-mouse (1:5,000; Invitrogen, AlexaFluor® 568 goat anti-mouse IgG1) were used besides DAPI for nuclear counterstaining. For the TMA, two replicate 1 mm-diameter cores were obtained. Three μm sections were deparaffinized, rehydrated, and quenched for endogenous peroxidase by incubating with 3%H_2_O_2_. For TRAP1 staining (1:3,000; Epitomics) epitope heat retrieval was performed by microwaving the slides in EDTA (0.01 M, pH 8.0). Samples were blocked with blocking solution (Ultra V Block; TA-015HP) for 7 min and then the primary TRAP1 antibody was added for one hour at room temperature. As a positive control, kidney tissue sections were used and for negative control, a rabbit immunoglobulin control. For enhancement, slides were incubated with primary antibody enhancer (Primary Antibody Enhancer; TL-015-PB) for 10 min followed by a HRP Polymer (HRP Polymer; TL-015-PH) for 15 min. Finally, slides were incubated with diaminobenzidin as a chromogen, counterstained with hematoxylin, dehydrated and mounted.

TRAP1 expression levels were determined using a scoring system based on the intensity of staining (0–3) compared to the negative control (0). Samples were examined by three independent observers, including a gynecological pathologist, whereby rescoring was conducted in samples with inconsistent scoring, leading to the following groups: negative (0); weak (1); moderate (2); and strong (3) staining. For statistical analysis classification into TRAP1-high and TRAP1-low was performed according to strong versus negative to moderate expression levels, respectively.

Staining for ERα (1:50; ERα, clone 1D5, mouse IgG1, Dako, Denmark) was performed using standard immunohistochemical techniques. The intensity patterns and nuclear positivity of ERα staining were analyzed by two independent co-workers, including a gynecological pathologist, applying a semi-quantitative scale of ImmunoReactive Score (IRS) including intensity of color reaction and percentage of positive cells. The intensity of reaction was scored as negative (intensity 0, <10% positive cells) or positive (intensity 1–3, >10% positive cells).

### Statistical analysis

Statistical analyses were performed using SPSS software version 19 (IBM Corporation, Armonk, New York, USA). Correlations between TRAP1-expression, ERα-expression, and clinicopathological parameters were assessed by T-tests, Chi-square tests, and Fisher’s exact tests as appropriate. Results were adjusted for multiple testing by the Bonferroni-Holm method [23]. To analyze ERα together with TRAP1 expression, patients were classified into four groups: i) ERα-/TRAP1-low; ii) ERα-/TRAP1-high; iii) ERα+/TRAP1-low; iv) ER+/TRAP1-high; For survival analyses and impact on chemotherapy response, the following combination pattern was included in the calculations: i) a group comprised of ERα-/TRAP1-low versus ii) a group comprised of the other three combinations. Impact on progression free survival and overall survival was determined by univariate and multiple Cox proportional-Hazards model analyses. Impact on chemotherapy response was determined by uni- and multivariate logistic regression models. In addition, the univariate impact was illustrated by Kaplan-Meier estimates whereby differences in survival were analyzed by the Log-rank test.

## Results

### Study population

Tumor tissues of 208 EOC patients were used. Median age at time of cytoreductive surgery was 56 years (range 18–85 years). All 9 (4%) patients with FIGO II EOC received optimal cytoreductive surgery as well as 137 (66%) of the 199 patients diagnosed with FIGO III and IV. Within the observation period 96 patients died and the median follow up was 51 months. The clinicopathological characteristics of the 208 EOCs classified as TRAP1-high or TRAP1-low are shown in Table 1. No significant differences were found for the clinicopathological parameters between the two groups (adjusted p-values).

**Table 1:** Characteristics of the study population

**Study population divided into TRAP1-low and TRAP1-high**				
**N = 208**	**TRAP1-low**	**TRAP1-high**		
**Characteristics**	**n = 93 (%)**	**n = 115 (%)**	**p**	**Adjusted p**
**Age**				
≤ 55 (n = 98)	49 (23.6)	49 (23.6)		
> 55 (n = 110)	44 (21.2)	66 (31.7)	0.148	
**Histology**				
Serous (n = 183)	76 (36.5)	107 (51.4)		
Non-serous^1^ (n = 25)	17 (8.2)	8 (3.8)	0.013	0.078
**FIGO**				
II (n = 9)	5 (2.4)	4 (1.9)		
III (n = 164)	70 (33.6)	94 (45.2)	0.526^2^	
IV (n = 35)	18 (8.7)	17 (8.2)		
**Grade** (1missing)				
Grade 1&2 (n = 55)	19 (9.2)	36 (17.4)		
Grade 3 (n = 152)	73 (35.3)	79 (38.2)	0.087	
**Residual tumor**				
no (n = 146)	64 (30.8)	82 (39.4)		
> 0 cm (n = 62)	29 (13.9)	33 (15.9)	0.697	
**Peritoneal Carcinomatosis**				
no (n = 64)	28 (13.5)	36 (17.3)		
yes (n = 144)	65 (31.5)	79 (38.0)	0.852	

^*1*^*Endometrioid (n = 9), mixed epithelial (n = 9), mucinous (n = 1), undifferentiated (n = 4), and clear-cell carcinoma (n = 2).*^2^Fisher’s Exact test.

### Immunohistochemical evaluation

**Figure 1:**
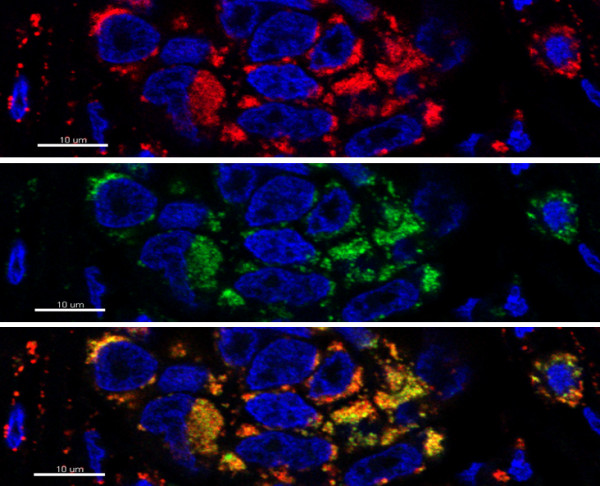
**Colocalization analysis of TRAP1 (green) and the mitochondrial marker COX5 (red) using double-immunofluorescence staining in an EOC sample.** The images show that both TRAP1 and COX5 are localized in the mitochondria. (Pictures were taken with the confocal microscope LSM700).

**Figure 2:**
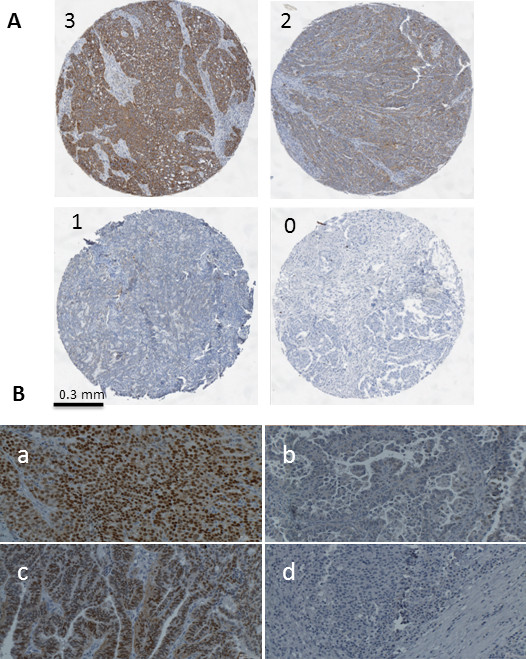
**A) Representative immunohistochemical staining of TRAP1 in four different EOC samples classified as TRAP1 negative (0); weak (1); moderate (2); and strong (3); surrounding stromal tissue showed no TRAP1 staining; B) Staining of ERα (a, c) highly positive (strong intensity of nuclear staining, >80% positive nuclei), (b) <10% of nuclei show weak staining, and (d) completely negative for nuclear ERα staining, were scored as ERα negative.** Pictures were taken using TissueFAXS (TissueGnostics; Vienna, Austria).

Initially, expression of the molecular chaperone TRAP1 in human ovarian cancer was determined by immunohistochemistry. 115 patients were classified as TRAP1-high (55.3%) and 93 patients as TRAP1- low (44.%), comprising moderate, low, and negative expression in 57 (27.4%), 25 (12.0%), and 11 (5.3%) samples, respectively. To proof the mitochondrial expression of the used antibody, a colocalization experiment was conducted, using the mitochondrial marker COX5 together with TRAP1. In Figure 1, an example of this double-staining is given, showing the mitochondrial localization of TRAP1 in an EOC tissue. On the TMA, tumor tissue showed a distinct specific intracytoplasmatic granular staining, reflecting the expected mitochondrial localization. To confirm the antibody specificity, a Western blot and staining of paraffin embedded agarose cell blocks of four cell lines (two with high, one with weak, and one with nearly no TRAP1 expression) have been conducted. The Western blot showed a perfect specificity (no additional bands besides the correct band at M_r_ ~ 75,000) of the TRAP1 antibody. A near perfect correlation of the Western band intensities to the staining intensities of the corresponding stainings of the agarose cell blocks was observed (supplementary data in additional file 1). Samples of studied ovarian cancer tissues with variable staining intensities are presented in Figure 2A. Tumor-surrounding stromal tissue showed no TRAP1 expression (Figure 2A).

The results of ERα immunohistochemistry analysis revealed 99 (48%) ERα negative and 109 (52%) ERα positive cases. Representative pictures of ERα staining are provided in Figure 2B. Table 2 describes the characteristics of ERα positive and negative patients. There was a significant correlation between ERα expression and the histological classification: tumors of non-serous histology were more likely to be ERα negative (adj. p = 0.045).

**Table 2:** **Study population divided into ERα negative (ERα**^**-**^**) and ERα positive (ERα**^**+**^**)**

**N = 208**	**ERα**^**-**^	**ERα**^**+**^		
**Characteristics**	**n = 99 (%)**	**n = 109 (%)**	**p**	**Adjusted p**
**Age**				
≤ 55 (n = 98)	55 (26.4)	43 (20.7)		
> 55 (n = 110)	44 (21.2)	66 (31.7)	0.026	0.080
**Histology**				
Serous (n = 183)	81 (38.9)	102 (49.0)		
Non-serous^1^ (n = 25)	18 (8.7)	7 (3.4)	0.009	**0.045**
**FIGO**^**2**^				
II (n = 9)	3 (1.4)	6 (2.9)		
III (n = 164)	76 (33.6)	88 (42.3)	0.158^2^	
IV (n = 35)	20 (9.6)	15 (7.2)		
**Grade** (1 missing)				
Grade 1&2 (n = 55)	18 (8.7)	37 (17.9)		
Grade 3 (n = 152)	81 (39.1)	71 (34.3)	0.009	0.054
**Residual tumor**				
no (n = 146)	66 (31.7)	80 (38.5)		
> 0 cm (n = 62)	33 (15.9)	29 (13.9)	0.289	
**Peritoneal Carcinomatosis**				
no (n = 64)	31 (14.9)	33 (15.9)		
yes (n = 144)	68 (32.7)	76 (36.5)	0.871	

^*1*^*Endometrioid (n = 9), mixed epithelial (n = 9), mucinous (n = 1), undifferentiated (n = 4), and clear-cell carcinoma (n = 2).*^*2*^Fisher’s Exact test.

To determine the relationship between ERα expression and TRAP1 expression, the Chi-square test was used. Ovarian cancer tissues expressing ERα were more likely to show high TRAP1 expression levels (67.0%, p < 0.001). Correspondingly, low TRAP1 expression levels were found in the majority of ERα negative tissues. Still, 42.2% of ERα negative samples presented high TRAP1 levels.

### Relevance of ERα and TRAP1 expression for chemotherapy response and patients’ survival

In Table 3A,B the impact of TRAP1, ERα, and the combined TRAP1/ERα expression pattern on OS and PFS, together with various clinicopathological parameters considered as potential prognostic factors, is presented. Univariate analyses identified age (in decades) (HR = 1.44; 95%CI [1.21-1.71]), FIGO stage (HR 1.96; 95%CI [1.25-3.06]), grade (HR 1.74; 95%CI [1.05-2.88]), residual tumor load (HR =2.35; 95%CI [1.54-3.61]), peritoneal carcinomatosis (HR = 3.01; 95%CI [1.75-5.16]), ERα (HR = 0.56; 95%CI [0.37-0.84]), TRAP1 (HR 0.63; 95%CI [0.42-0.94]), and the combination pattern ERα/TRAP1 (HR = 0.49; 95%CI [0.32-0.75]) to be significantly associated with OS. For PFS, the parameters age, FIGO-stage, grade, residual tumor load, and peritoneal carcinomatosis showed significant impact (Table 3B).

**Table 3:** Multiple Cox and logistic regression analyses for (A) overall survival, (B) progression free survival, and (C) chemotherapy response of clinicopathological parameters, ERα, TRAP1, and the ERα/TRAP1 expression pattern

**A) Overall survival**								
**N = 208**	**Univariate**		**Multivariate**					
			**ERα**		**TRAP1**		**Combination**	
**Characteristics**	**HR (CI95%)**	**p**	**HR (CI95%)**	**p**	**HR (CI95%)**	**p**	**HR (CI 95%)**	**p**
Age (per decade)	**1.44 (1.21-1.71)**	**<0.001**	**1.40 (1.17-1.68)**	**<0.001**	**1.42 (1.18-1.71)**	**<0.001**	**1.50 (1.24-1.82)**	**<0.001**
Histology (non-serous vs serous)	1.05 (0.57-1.92)	0.874	1.20 (0.64-2.27)	0.555	1.22 0.65-2.29)	0.545	1.33 (0.70-2.52)	0.381
FIGO (IV vs III vs II)	1.96 (1.25-3.06)	0.003	1.39 (0.85-2.25)	0.187	1.38 (0.85-2.24)	0.188	1.39 (0.86-2.27)	0.176
Grade (3 vs 1,2)	1.74 (1.05-2.88)	0.032	0.98 (0.58-1.67)	0.951	1.34 (0.67-1.92)	0.631	0.96 (0.57-1.64)	0.893
Residual tumor (yes vs no)	**2.35 (1.54-3.61)**	**<0.001**	**1.87 (1.21-2.89)**	**0.005**	**1.79 (1.16-2.77)**	**0.009**	**1.80 (1.17-2.78)**	**0.008**
Peritoneal Carcinomatosis (yes vs no)	**3.01 (1.75-5.16)**	**<0.001**	**2.75 (1.56-4.84)**	**<0.001**	**2.45 (1.38-4.32)**	**0.002**	**2.68 (1.51-4.74)**	**0.001**
ERα (1/2/3 vs 0)	**0.56 (0.37-0.84)**	**0.005**	**0.51 (0.34-0.77)**	**0.002**				
**TRAP1 (3 vs 0/1/2)**	**0.63 (0.42-0.94)**	**0.025**			**0.65 (0.43-0.99)**	**0.044**		
Combination ERα/TRAP1*	**0.49 (0.32-0.75)**	**0.001**					**0.41 (0.27-0.64)**	**<0.001**
**B) Progression free survival**								
**Characteristics**	**HR (CI95%)**	**p**	**HR (CI95%)**	**p**	**HR (CI95%)**	**p**	**HR (CI 95%)**	**p**
Age (per decade)	**1.21 (1.06-1.38)**	**0.041**	1.14 (0.99-1.31)	0.056	1.15 (1.00-1.32)	0.055	**1.17 (1.02-1.35)**	**0.027**
Histology (non-serous vs serous)	0.91 (0.55-1.49)	0.701	1.23 (0.72-2.11)	0.453	1.25 (0.73-2.15)	0.416	1.25 (0.72-2.14)	0.428
FIGO (IV vs III vs II)	**2.48 (1.69-3.62)**	**<0.001**	**1.85 (1.22-2.79)**	**0.004**	**1.91 (1.26-2.89)**	**0.002**	**1.91 (1.26-2.89)**	**0.002**
Grade (3 vs 1,2)	**1.49 (1.03-2.17)**	**0.035**	0.93 (0.62-1.39)	0.721	0.95 (0.64-1.41)	0.794	0.89 (0.59-1.33)	0.559
Residual tumor yes vs no)	**1.99 (1.45-2.75)**	**<0.001**	**1.56 (1.11-2.18)**	**0.010**	**1.55 (1.10-2.17)**	**0.011**	**1.56 (1.11-2.18)**	**0.010**
Peritoneal Carcinomatosis (yes vs no)	**2.96 (2.01-4.37)**	**<0.001**	**2.98 (1.95-4.55)**	**<0.001**	**2.92 (1.91-4.47)**	**<0.001**	**3.00 (1.96-4.60)**	**<0.001**
ERα (1/2/3 vs 0)	0.84 (0.62-1.15)	0.279	0.80 (0.58-1.11)	0.185				
TRAP1 (3 vs 0/1/2)	0.87 (0.63-1.18)	0.367			0.87 (0.63-1.20)	0.398		
Combination ERα/TRAP1*	0.79 (0.56-1.12)	0.188					0.70 (0.48-1.02)	0.061
**C) Chemotherapy response**								
**Characteristics**	**HR (CI95%)**	**p**	**HR (CI95%)**	**p**	**HR (CI95%)**	**p**	**HR (CI 95%)**	**p**
Age (per decade)	**1.33 (1.02-1.74)**	**0.036**	**1.42 (1.06-1.9)**	**0.019**	**1.43 (1.06-1.91)**	**0.019**	**1.57 (1.15-2.15)**	**0.005**
Histology (non-serous vs serous)	0.74 (0.26-2.08)	0.567	0.92 (0.30-2.83)	0.889	0.93 (0.30-2.83)	0.893	0.88 (0.28-2.73)	0.824
FIGO (IV vs III vs II)	**2.06 (1.02-4.15)**	**0.044**	1.59 (0.73-3.49)	0.241	1.65 (0.76-3.61)	0.207	1.67 (0.75-3.72)	0.205
Grade (3 vs 1,2)	1.17 (0.56-2.44)	0.683	0.72 (0.32-1.64)	0.437	0.76 (0.33-1.72)	0.505	0.56 (0.23-1.33)	0.188
Residual tumor (yes vs no)	**1.96 (1.01-3.80)**	**0.046**	1.35 (0.67-2.75)	0.411	1.43 (0.71-2.91)	0.318	1.36 (0.66-2.81)	0.408
Peritoneal Carcinomatosis (yes vs no)	**3.01 (1.32-6.85)**	**0.009**	**3.04 (1.25-7.36)**	**0.014**	**2.89 (1.19-7.01)**	**0.018**	**3.34 (1.34-8.36)**	**0.009**
ERα (1/2/3 vs 0)	0.62 (0.33-1.17)	0.138	0.53 (0.26-1.06)	0.071				
TRAP1 (3 vs 0/1/2)	**0.53 (0.28-1.00)**	**0.050**			**0.48 (0.24-0.96)**	**0.037**		
Combination ERα/TRAP1*	**0.38 (0.19-0.75)**	**0.005**					**0.24 (0.11-0.54)**	**< 0.001**

*ERα and/or TRAP1 high vs ERα- and TRAP1 low.

In a next step, multivariate Cox proportional-hazards regression analyses of patients’ OS and PFS, based on clinicopathological factors together with either i) TRAP1 expression, ii) ERα expression or iii) the combination pattern, was performed. Multivariate analyses identified ERα and TRAP1 to have an independent impact on OS (HR = 0.51; 95%CI [0.34-0.77] and HR = 0.65; 95%CI [0.43-0.99]), respectively). The TRAP1/ERα-combination pattern was an even stronger significant independent prognostic factors for OS (HR = 0.41; 95%CI [0.27-0.64]) (Table 3A). Age and peritoneal carcinomatosis showed a comparably strong influence on OS. FIGO stage, residual tumor load and peritoneal carcinomatosis had a significant impact on PFS.

In Table 3C the impact of TRAP1, ERα, and the combined TRAP1/ERα expression pattern on the response to first-line chemotherapy is shown. The risk to be a non-responder was significantly lower in patients with high TRAP1 expression compared to patients with low TRAP1 expression, both, in a univariate (HR = 0.53; 95%CI [0.28-1.00]) and a multivariate analysis (HR = 0.48; 95%CI [0.24-0.96]). A similar but not significant impact was found for ERα positivity in a univariate (HR = 0.62; 95%CI [0.33-1.17]) and a multivariate analysis (HR = 0.53; 95%CI [0.26-1.05]). Combining both factors, halves the observed risk to be a non-responder compared to the risk of patients with TRAP1 high or ERα positive tumors, in the univariate (HR = 0.38; 95%CI [0.19-0.75]) and multivariate (HR = 0.24; 95%CI [0.11-0.54]) analysis, indicating an additive effect of both markers.

**Figure 3:**
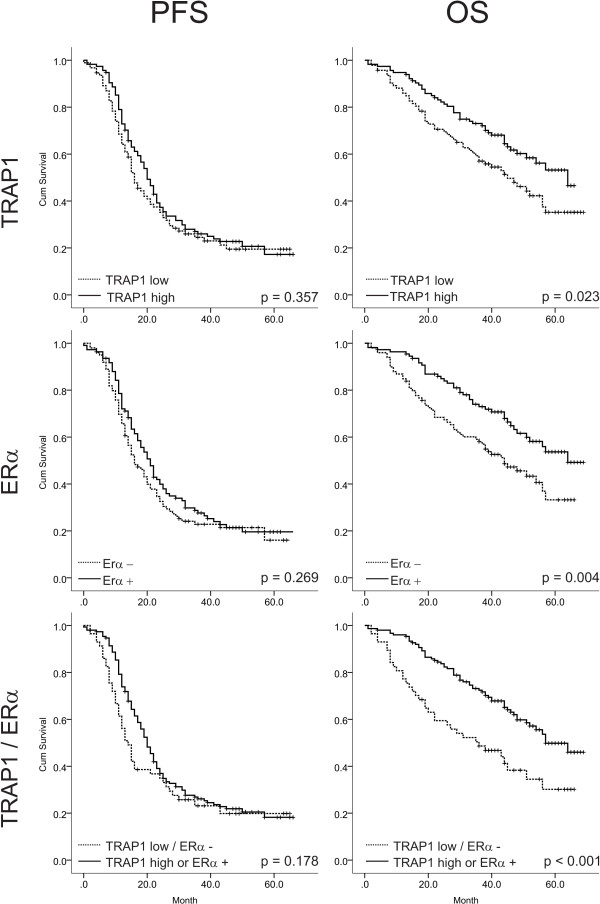
Kaplan-Meier estimates of the impact of TRAP1, ERα, and the combination pattern on PFS and OS (p values determined by the log-rank test).

**Figure 4:**
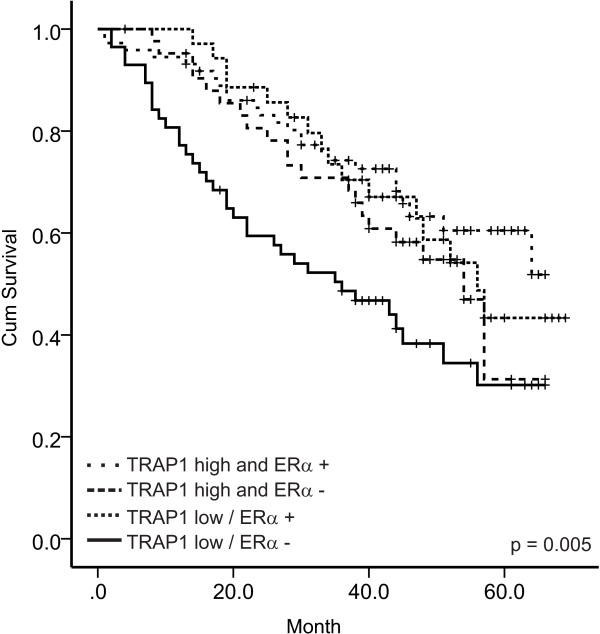
**Kaplan-Meier estimates comparing the impact of all four groups of the combined TRAP1-ERα pattern on OS.** Black line: ERα negative/TRAP1-low; dotted black line: ERα negative/TRAP1-high; grey line: ERα positive/TRAP1-high; dotted grey line: ERα positive/TRAP1-low.

Figure 3 shows the univariate impact of ERα, TRAP1, and the combination pattern on PFS and OS as estimated by Kaplan Meier plots. Stratifying the patients into two groups according to their combined TRAP1/ERα expression level yields the strongest predictive factor, which was also seen from the corresponding multiple Cox proportional-hazards regression models (data not shown). Patients presenting with a double negative expression pattern (TRAP1-low/ERα-) at time of cytoreductive surgery appear to have a considerably decreased OS (p < 0.001) and PFS (p = 0.005) compared to patients with positive expression levels of both or one of the examined parameters (Figure 3). Further stratification of group 2 into TRAP1high/ERα- (group 2a) and TRAP1low/ERα + (group 2b), and TRAP1high/ERα + (group 2c) did not improve this predictive capacity, as shown for OS in Figure 4 (differences not significant).

## Discussion

We have evaluated the expression of ERα in EOC together with the expression of TRAP1, that has been described to be up-regulated *in vitro* in ER positive ovarian cancer cells exposed to estrogen [9]. To our knowledge, this is the first study evaluating the impact of TRAP1 expression on patients’ outcome in a large prospectively collected cohort of more than 200 patients with EOC and the first study evaluating the combined prognostic impact of TRAP1 and ERα.

Correlation of ERα with TRAP1 was significant, whereby ERα positive tumors presented significantly higher expression levels of TRAP1. However, high TRAP1 levels were also found in 42% of ER negative tumors, revealing two independent but interconnected parameters i) ERα, described to play a dominant role in ovarian cancer [24,25] and ii) TRAP1, a mitochondrial chaperone, selectively up-regulated in tumor cells [19] and up-regulated by estrogen [10].

ERα expression was not significantly associated with stage and grade, which is in accordance with the study of Hecht *et al*. [26]. Additionally, we found no significant correlation with age, peritoneal carcinomatosis, and residual tumor load. Tumors with non-serous histology were more likely to be ERα negative (adj. p = 0.045) compared to serous histology, which is in accordance with the findings of Lee *et al.*[15]. Studies on ERα expression and survival are inconsistent [12-16,18,27]. The results of this study show a significantly longer OS for ERα positive patients in a multiple Cox regression analysis. TRAP1 expression showed a significant and independent impact on chemotherapy responder status and on patients’ OS. Combining TRAP1 and ERα expression, patients with ERα negative and TRAP1-low expressing tumors, compared to all other combinations, showed a 2.44-fold higher risk to die. This indicates a subcohort of EOC patients presenting with a low TRAP1 expression and a negative estrogen receptor status exhibiting a worse prognosis.

Up to now, only one study has addressed TRAP1 protein expression in human ovarian cancer tissue in regard to therapy response. Walker et al. have described significantly increased expression levels of TRAP1 in Letrozole-sensitive tumors if compared to resistant tumors, suggesting that endocrine responsiveness might be predictable with the help of estrogen regulated genes and their expression levels [10]. Still, the association of TRAP1 expression in patients receiving Letrozole therapy and response has not been investigated in a multivariate model, and thus, the independent impact of TRAP1 on response to therapy remains unclear. Therefore, we focused on the predictive independent impact of TRAP1 and ERα on response to standardized chemotherapy and on patients’ outcome. In accordance with the study of Walker e*t al*. [10], our results show that high TRAP1 expression is positively associated with therapy-response. Patients with high TRAP1 expression have a two-fold higher response-rate to first-line chemotherapy. Similarly, patients with ERα positive tumors show a two-fold higher response rate, but only as trend when corrected for clinicopathological parameters. For the combined expression pattern of TRAP1 and ERα, a more than four-fold higher risk to be a chemotherapy non-responder for patients with TRAP1 weak and ERα negative tissues could be observed (p < 0.001). Most of the current knowledge on TRAP1 has been derived by cell-line models and mouse-models [28,29]. This is the first study investigating TRAP1 in the complex biology of EOC patients treated with standardized chemotherapy. In cell line models investigating Cisplatin resistance only weak differences in the TRAP1 expression (1.1-1.2 fold) between resistant and sensitive cells were observed [8]. The authors therefore concluded that TRAP1 might not lead to resistance in an *in vitro* model.

As shown with small interfering RNA gene-silencing of TRAP1 in a lung cancer cell line and re-expression in a breast cancer cell line, TRAP1 expression seems not to be associated with apoptosis [30]. This is in conflict with a variety of studies, proposing anti-apoptotic and anti-oxidative functions of TRAP1 [19,20,29]. As shown above for human EOC patients, high TRAP1 expression – as determined by immunohistochemistry – reveals significantly better response to chemotherapy and a longer OS. To better understand the conflicting data within different *in vitro* models and between some *in vitro* models and our *in vivo* survival analyses, the role of TRAP1 in EOC needs to be further elucidated.

## Conclusion

As only few studies are available on the role of TRAP1 in EOC, this study enhances the knowledge upon the crosstalk between TRAP1 and ERα in clinical samples. However, caution is needed in the biological interpretation of TRAP1’s role in human EOC. Indeed, several reports suggested that TRAP1 is involved in protection from apoptosis. Thus, the finding that EOC patients with high TRAP1 expression are characterized by an advantage in chemotherapy response and overall survival would suggest a converse involvement of TRAP1 in an *in vivo* setting, e. g. that TRAP1 is a (surrogate) marker for stressed, thus apoptosis prone, tumor cells. This would explain the positive impact of high TRAP1 expression on chemotherapy response and overall survival. In such a perspective, further studies in either EOC cell lines or human EOC series are needed to understand the biological and clinical role of HSP90 chaperones in ovarian carcinogenesis.

## Conflicts of interests

The authors have no conflicts of interest to declare.

## Supplementary Material

Additional file 1 Supplementary data. Click here for file
