# Supplementary material for: Role of TRAP1 and estrogen receptor alpha in patients with ovarian cancer -A study of the OVCAD consortium
Source: Mol Cancer. 2012 Sep 14;11:69. doi: 10.1186/1476-4598-11-69 (PMC3533746; doi:10.1186/1476-4598-11-69)

Supplementary data to

**Role of TRAP1 and estrogen receptor alpha in patients with ovarian cancer -**

**A study of the OVCAD consortium**

**Material and Methods**

**Cell culture:** Four cell lines were selected according to the TRAP1 expression levels determined by microarray analysis (unpublished data). HeLa (cervical cancer), and MCF7 (breast cancer) as TRAP1 high expressing cell lines; COR-L24 (small cell lung cancer, growing as aggregates in suspension) and MM231 (breast cancer) as TRAP1 low expressing cell lines; all cell lines were cultured according to standardized protocols.

**Cell lysate preparation and western blot analysis:** Cell lysates were prepared using 200 µl lysis buffer per sample (1% Triton X-100, 10mM Tris pH 7.4, 150mM NaCl, 5mM EDTA, protease/phosphatase inhibitor cocktail, Roche). The protein concentrations of the cell lysates were determined using Pierce® BCA Protein Assay Kit (Thermo Scientific) as previously described by Yotova et al ([Yotova *et al*, 2011](#_ENREF_1)). Twenty micrograms of normalized samples were immunoblotted and probed with the following primary antibodies: TRAP1 (1:10,000, Epitomics, USA) and ß-Actin (1:4,000, Santa Cruz, USA) before incubation with peroxidase-conjugated secondary antibodies (Pierce Chemical Co, USA). Western blot analysis was performed as previously described by Yotova et al ([Yotova *et al*, 2011](#_ENREF_1)). Bound antibodies were detected by enhanced chemiluminescence plus western blotting detection system (Amersham Pharmacia Biotech, Inc., NJ, USA) and exposed to X-ray films (GE Health Care, UK).

**Cell block preparation and immunohistochemistry:** Paraffin-embedded agarose cell blocks were made from each of the four cell lines to reach a starting situation comparable to the paraffin embedded EOC tumor tissues. Briefly, cells were fixed in 4% formalin for 30 minutes at room temperature, washed, and the cell pellet was mixed with melted hand-hot agarose to obtain agarose cell blocks which were subsequently embedded in paraffin. After cutting, deparaffinization, and antigen retrieval (same method as used for the tissue microarrays) of the sections, cells were stained with TRAP1 antibody (1:3,000, Epitomics, USA) exactly as described in the material and methods section of the manuscript.

**Results**

Immunohistochemical stainings of TRAP1 on paraffin embedded agarose cell blocks of four cell lines are depicted in Fig. 1A. To test the specificity of the used monoclonal TRAP1 antibody and to determine the expression of TRAP1 in these cell lines, Western blot analysis was performed. The protein expression correlated roughly to the microarray data and the Western blot showed a perfect specificity for TRAP1 (M_r_ ~ 75,000), proved by the absence of additional bands.

The immunohistochemical staining intensities of TRAP1 in HeLa and MCF7 cells were high, matching to the intense bands of TRAP1 expression obtained by Western blot analysis (Supp. Fig. 1B). COR-L24 cells show virtually no TRAP1 staining and only low TRAP1 staining intensity could be observed in MM 231 cells. The results in Supp. Fig. 1B show that these intensity levels match to the expression levels obtained by Western blot analysis.

**References**

Yotova IY, Quan P, Leditznig N, Beer U, Wenzl R, Tschugguel W (2011) Abnormal activation of Ras/Raf/MAPK and RhoA/ROCKII signalling pathways in eutopic endometrial stromal cells of patients with endometriosis. *Hum Reprod* **26**(4)**:** 885-97


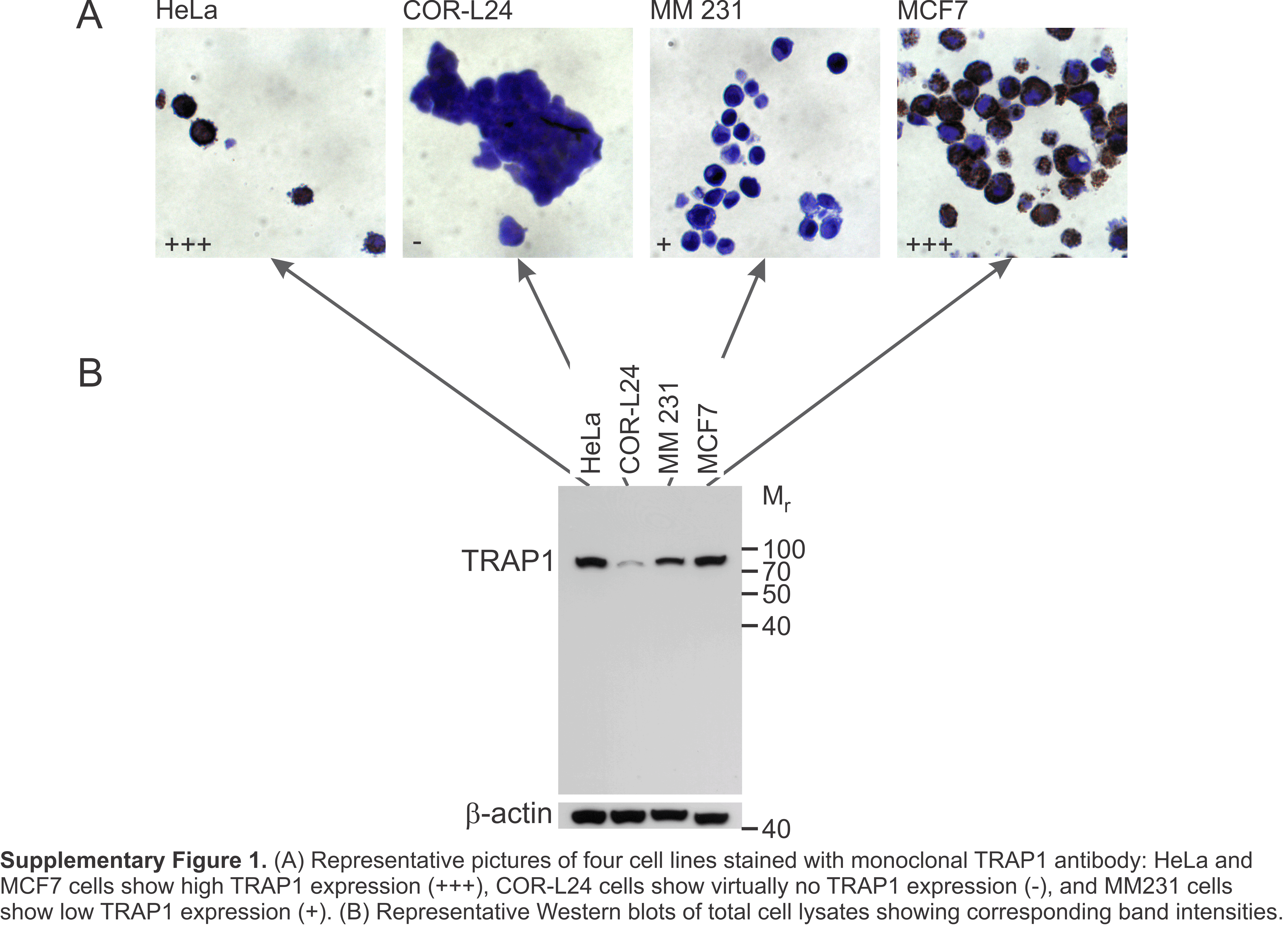

Supplement: Additional file 1 — Supplementary data. [file 1476-4598-11-69-S1.docx]
